# 

**Published:** 1877-10

**Authors:** 


### CONTENTS.

PAGE.

**CONTRIBUTIONS.**

How Boys are made Bad. Ben. H. Pratt ........281
One Health, One Disease. P. H. Hayes, M. D. .......283
Nurses and Neighbors. Thos. K. Beecher ............285

**MEDICAL ITEMS AND NEWS.**

Chloral Hydrate—Dr. Dublanchet—The Plague—Anthrax Maligna—While there is Life, there is Hope—Pulse by Telegraph—Photographs of Bacteria—Mustard Paper—Turpentine Bath—Diphtheria—Tooth-Filling—Gout-Paper—Atony of the Bladder—Urticaria—Hæmorrhoids—Itching—Sciatica—Constipation—Lesions from Alcohol—Mistura Guaiaci Viridis—Chronic Bronchitis—Croton Oil to the Skin—Camphor Vapor Baths—Typhoid Fever—Uterine Hæmorrhages—Small Pox Poison—External use of Chloral Hydrate—Antiseptic Vinegar — Cosmoline — Amenorrhœa and Obesity—Insomnia—Subcutaneous Injection of Blood—Chloroform Narcosis—Dangers of Ether—Homœopathy—Pityriasis Capitis—Cancer of Stomach—Value of Repeated Small Doses...287 to 296

**OUR CORRESPONDENCE.**

Four Letters with Replies.................................297-298

**HOUSEHOLD HINTS AND RECIPES.**

To Restore Gilt—To Remove Printing Ink—Offensive Feet—To Keep Meat Fresh—Cure for Soft Corns—French Blacking—Frosted Bouquets—To Remove Tin from Tinned Copper—To Clean Straw Matting—Hard Corns—To Remove Stains from White Marble—To Fix Ink—Carbon Bisulphide Lamp—To Clean Cane-Bottom Chairs—To Cure Hams—To Cure Beef and Pork—Conservatory on the House-Top—Small-Pox—To clean Oil Paintings—To Clean Pictures—To Clean Paper Hangings—Vegetable Growth, Mosses, etc., on Walls—Chloride of Lime as a Disinfectant—Guarana Chocolate—Shaving Soap—To Paint Magic Lantern Slides—Disinfectant—Cucumber Pickles—To Pickle Cauliflower—Pickled Cabbage—Green Pickles--Chocolate Caromels--Molasses Candy 299-302

**EDITORIAL.**

Children's Eyes ........................................ 303
Ectropium ........................................ 303
Our Inheritance ........................................ 304
Clothing ........................................ 305

**EDITORIAL CHIT-CHAT.**

Sweet Forget Me Not—An Artist, B. Browne--Pleasanton's Humbug—Our Advertisers—Garden Statues—The Telephone—Florists—Pay Your Subscription—Brigham Young—Autumn Leaves—A Ghost—The Turks—Jim Crow—Popular Science Monthly—Street Frights—Home—A Contribution—Spitting—That New York P. M—Horse-Racing—A Flea—My Nervous Patient........306 to 308

